# Identification and control of a multiplace hyperbaric chamber

**DOI:** 10.1371/journal.pone.0200407

**Published:** 2018-08-03

**Authors:** Luis Gracia, Carlos Perez-Vidal, Jose M. de Paco, Luis M. de Paco

**Affiliations:** 1 Instituto IDF, Universitat Politècnica de València, Camino de Vera s/n, 46022 Valencia, Spain; 2 Department of Systems Engineering and Automation, Miguel Hernandez University, Elche, Spain; 3 European Organization for Nuclear Research, CERN, Geneve, Switzerland; Shandong University of Science and Technology, CHINA

## Abstract

This work presents the automation of a multiplace hyperbaric chamber. It includes the system modeling, identification, controller calculation and system validation. With the proposed approach a good pressure profile tracking and repeatability are achieved. Moreover, the proposed automation includes the implementation of powerful treatment tools such as Pause and Alleviation procedures. The control system implemented is based on a special zero-pole cancellation regulator. Experimental results are provided to illustrate the behavior of the automated chamber. It is important to remark that the chamber automated in this work is being successfully used in a real hospital since 2015 treating more than 40 patients per day, five days a week.

## Introduction

Hyperbaric oxygenation is a medical treatment in which the patient is progressively subjected to a pressure greater than the sea level atmospheric pressure, usually between 1.5 and 2.5 bar. The patient then breathes pure oxygen during a controlled period of time. This oxygen can be the gas used to compress the chamber or can be supplied to the patient through a mask. After the treatment, the patient is progressively subjected to a lower pressure (decompressed) until the atmospheric pressure is reached again.

A hyperbaric chamber (HC) is a vessel, usually made of steel, in which the pressure can be raised by compressing the air, or sometimes by compressing oxygen. Basically, HCs can be classified in two types depending on their size and use: *monoplace* HCs, which are used only by one person; and *multiplace* HCs, which are used by a group of people receiving the same treatment.

**Fig 1:**
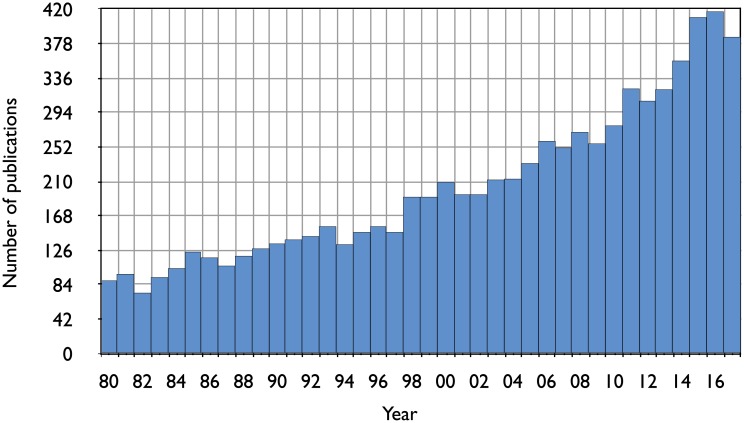
Number of papers related to hyperbaric oxygen (PubMed.gov search with keyword ‘hyperbaric oxygen’) between years 1980 and 2017.

Although hyperbaric medicine has been used for decades, this medical area is gaining in popularity [1, 2], as evidenced by the rise in the number of papers related to hyperbaric oxygen published in journals between 1980 and 2016, see Fig 1. This information has been simply obtained by searching in PubMed.gov using the keywords: “hyperbaric oxygen”.

Hyperbaric medicine experimented an important growth during the 1960s and 1970s [3] and due to the long lifetime of HCs, many of them remain still working in hospitals with a low level of automation (e.g., they are manually controlled by using mechanical sticks or potentiometers to open a valve in order to increase the pressure). This work is aimed to old HCs that need to be automated and new ones manufactured by small or medium companies.

The accepted uses of hyperbaric oxygen therapy as defined by the European Underwater and Baromedical Society and the Undersea and Hyperbaric Medical Society (USA) are: air or gas embolism; carbon monoxide poisoning; cyanide poisoning; clostridial myositis and myonecrosis (gas gangrene); crush injury, compartment syndrome and other acute traumatic ischemias; decompression sickness and many others [4, 5].

Automation techniques are now being used in almost all areas of daily life, from domestic gadgets to the heaviest industries. In this sense, big manufacturers are including automation technologies in their products. This is the case of Dragerwerk AG and Haux-Life in Europe, Perry Baromedical Corporation, Hyperbaric Technologies Inc., Sechrist Industries and Reimers Systems in USA, and Kawasaki Heavy Industries and Nakamura in Japan. But HCs manufactured by SMEs (Small or Medium Enterprises) are not automated yet due to their lack of R&D resources. This paper is aimed to those companies without R&D departments and it gives to them a guideline for HC’s automation.

It is important to remark that this work is focused on HCs’ automation. Although some companies offer commercial solutions on this matter as mentioned above, to the best of the authors’ knowledge there is no published literature (peer-reviewed journal papers) addressing this issue, hence the work presented here. In particular, after a through search in relevant databases like Web of Science, Scopus and Google Scholar no research paper dealing with HCs’ automation has been found. Only some patents have been reported on this issue [6–8], although all of them present vague descriptions and give no details for the modeling, identification and control algorithms. In contrast to these patents, this work is not only novel but also *useful* since all the details required to automate an existing manual HC are provided.

The structure of the paper is as follows. Next section introduces the methods used in this work to automate a HC, while Section 3 presents the results obtained with the proposed approach. Then, Section 4 discusses several relevant issues about the use of the automated HC. Finally, Section 5 shows the conclusions.

## Methods

### Multiplace hyperbaric chamber

**Fig 2:**
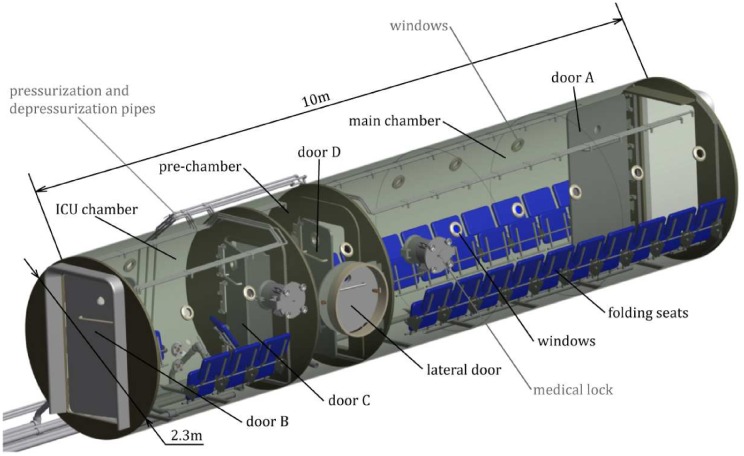
Structure, distribution and size of the MEDIBAROX multiplace and multi-compartment HC at Perpetuo Socorro Hospital in Alicante (Spain). Patients sit in the blue chairs.

The HC used in this work is the MEDIBAROX multiplace and multi-compartment chamber, see Fig 2. This chamber has three compartments: a main chamber, an Intensive Care Unit (ICU), and a pre-chamber between them. The chamber has a capacity for 25 seated people and ten gurneys or four ICU beds. The pre-chamber allows the access to the main chamber or the ICU from outside. The aim of the pre-chamber is to make possible the movement of patients and medical staff between compartments at different pressures, and to be able to introduce a doctor inside the chamber to treat a patient or to take out a patient in case of emergency.

**Fig 3:**
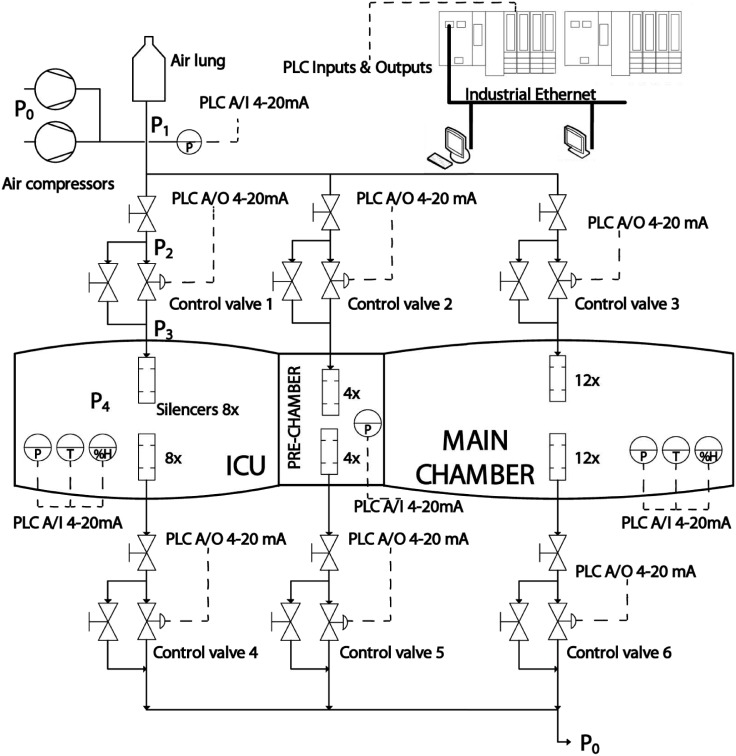
Multiplace chamber’s electromechanical scheme.

In order to automate this HC, the set-up given by the electromechanical scheme in Fig 3 has been used, where PLC stands for Programmable Logic Controller, A/O for analog output and A/I for analog input. There are three different circuits to pressurize each compartment of the chamber. Each circuit has a control valve and two safety valves. The air goes inside the chamber through pneumatic silencers to reduce noise. The decompression circuit is equivalent to the pressurization circuit. The Programmable Logic Controllers (PLCs) are the electronic devices in charge of controlling the chamber. They read the sensors’ analog signals and control the analog and digital actuators. The PLC communicates with a computer and a Human Machine Interface (HMI) via Industrial Ethernet to share information of the process.

Sensors and actuators used in this work are described below. All the components have been specially chosen to be used in oxygen enriched environments (although the chamber is pressurized with air, an oxygen escape could cause a hazardous situation). This equipment consists of: 6 pressure sensors, S-11 model (0 to 10 bar in a 4-20mA range), by WIKA (2 sensors per compartment in a redundant configuration); 3 temperature and humidity hair sensors, SEM165 by Status, especially designed for hyperbaric atmospheres; 6 flow control valves (2” size) manufactured by Metalwork, Skillair REG-300 model; 2 screw type air compressors, Atlas Copco ZT/ZR 110-900 VSD model; an air lung; a UPS (uninterruptible power supply) device; 2 Siemens PLCs ET-200S IM151-8 with distributed I/O (PLCs has been chosen according to ATEX Directive 94/9/EC.); a HMI SIMATIC MP 377 12 inch touch panel; and a computer with a Siemens WinCC Scada software.

### Procedures during a treatment

The automation of a multiplace chamber makes it easier to manage the procedures involved with going into and out of the chamber, which are described below [9]:

- *Lock-In Operations*: Personnel entering the main chamber or the ICU chamber go into the pre-chamber and close and lock the lateral door. The pre-chamber has to be pressurized at a rate controlled by the system to equalize, but not to exceed 2.25 bar per minute. The system shall record the time when the pressurization begins in order to determine the decompression schedule for the occupants when they are ready to leave the chamber. When the pressure levels in the pre-camber and ICU chamber are equal, door C (which was closed at the beginning of the treatment) should be open. Note that, door D remains closed all the time.
- *Lock-Out Operations*: To exit the ICU chamber, the personnel enter again the pre-chamber and they close and lock door C. When ready to ascend, the Diving Supervisor is notified and the required decompression schedule is selected and executed. Constant communications are maintained with the inside personnel to ensure that a seal has been made on door C. Pre-chamber depth is controlled throughout decompression by the system.

Once the patient is placed inside a HC, he or she performs the *Valsalva manoeuvre* to compensate the pressure at both sides of the tympanic membrane (ear drum) in order to avoid a *barotrauma* injury. The Valsalva manoeuvre is performed by slightly forcing exhalation against a closed airway, usually closing the mouth and pinching the nose. The air goes through the eustachian tubes to pressurize the internal side of the tympanic membrane. To avoid barotrauma impact, patients are previously trained to perform Valsalva manoeuvre (the medical staff uses an endoscopic camera during the training process to guarantee that the patient is able to compensate the pressure), although the impact of pressure increase is not completely overtaken.

**Fig 4:**
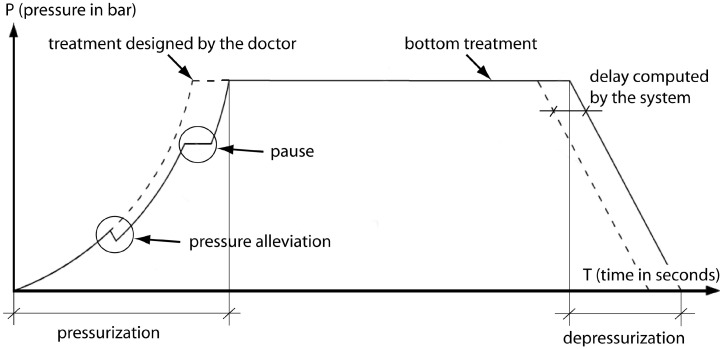
HBO2 events and steps during a sample treatment.

The most important procedures carried out in the chamber (based on the number of times applied) are Alleviations and Pauses. These procedures are basic but extremely important to avoid barotrauma in patients. They are performed when the Diving Supervisor detects that a patient feels some earache (patients have told to communicate any earache when they happen). Fig 4 shows a non-linear pressurization profile (*Smooth Ride* type), in which an Alleviation and a Pause are online introduced by the Diving Supervisor. These two procedures modify the moment in which the chamber achieves the bottom treatment and, for this reason, a delay computed by the system is applied to accomplish with the initial specifications indicated by the doctor.

The Alleviation procedure is performed during the compression process. In this case, a decompression profile is loaded to the decompression regulator which decreases the pressure a determined value following a determined decompression ratio. For example, if pressure is 1.45 bar at the moment of starting the Alleviation process, the alleviation value is 0.05 bar, and the decompression ratio is 0.1 bar/minute, the regulator will decrease the chamber until 1.40 bar in 30 seconds. When the Alleviation is finished, it is possible to carry out another alleviation or to carry on with the treatment.

The Pause procedure consists on a steady state in the reference profile in order to keep the pressure constant. When the pause procedure has started, the reference is stopped and the pressure is kept constant until the pause finishes, which is based on the Diving Supervisor criteria. Subsequently, the pressurization goes on from that point.

### Chamber modeling and identification

In multi-compartment chambers, it is possible to have different treatments running at the same time, see Fig 3. This section presents the mathematical modeling of the main chamber, but the process can be easily replicated for the ICU chamber and the pre-chamber.

#### Control valve modeling

The modeling of the air flow through a hole with a variable area is a highly non linear function which mainly depends on the input and output pressures and the size of the hole. This model is defined in different ways in technical literature [10–13] depending on the accuracy level.

The control valves used are pneumatically actuated. When the control pressure over the diaphragm changes, the piston rod slides and the valve opening size changes.

The equations to calculate the flow of compressible fluids through control valves are described in [11]. The volumetric flow in normal conditions (0°C and 1 bar) through the valve (incompressible and choked flow without attached fittings) is given by expressions (1) to (5), where:

- *q* is the volumetric flow rate (m^3^/h).
- *C*_*v*,*max*_ is the valve flow coefficient (m^3^/h). This was introduced by [14] and it is usually provided by the valve manufacturer. *C*_*v*_ (m^3^/h) depends on the opening of the valve. *C*_*v*_ is 0 when the valve is closed and *C*_*v*,*max*_ when it is completely open. This variation allows the valve to regulate the flow. “*a*” (dimensionless) value of (1) is the valve opening (a value between zero and one).
- *T* is the absolute inlet temperature (Kelvins).
- *G*_*g*_ is the gas specific gravity (ratio of density of flowing gas to density of air with both at standard conditions), which is considered in [11] to be equal to the ratio of the molecular weight of gas to molecular weight of air.
- *F*_*p*_ is the piping geometry factor (dimensionless).
- *F*_*γ*_ is the specific heat ratio factor (dimensionless) and *γ* is the gas specific heat ratio (dimensionless). Both are physical constants and can be found in [11].
- *Y* is the gas expansion factor (dimensionless).
- *x* is the pressure drop ratio (dimensionless). Limit *x* = *F*_*γ*_ ⋅ *x*_*T*_.
- *x*_*T*_ is the pressure differential ratio factor of a control valve without attached fittings at choked flow (dimensionless).
- *Z* is the gas compressibility factor (dimensionless).
- Δ*P*_*v*_ is the differential pressure between upstream and downstream pressure taps (*P*_2_ − *P*_3_ in kPa).
- *P*_0_ is the atmospheric pressure (kPa).
- *P*_1_ is the output pressure of the air compressors (kPa).
- *P*_2_ is the upstream pressure (kPa).
- *P*_3_ is the downstream pressure (kPa).
- *P*_4_ is the pressure inside the chamber (kPa).
- *P* is the incremental pressure inside the chamber (kPa), therefore Δ*P* = Δ*P*_4_.

Note that *P*_1_, *P*_2_, *P*_3_ and *P*_4_ are represented in the electromechanical scheme of Fig 3.

Gathering and simplifying expressions from (1) to (5) as indicated in [10], Eq (6) is obtained, where $C_{1}=417\cdot C_{v\;\;max}\cdot F_{P}\cdot \sqrt{P_{2}}$ and $C_{2}=\frac{-417\cdot C_{v\;\;max}\cdot F_{P}}{2.14\cdot \gamma \cdot F_{\gamma }\cdot x_{T}\cdot \sqrt{G_{g}\cdot T\cdot Z}}\cdot \frac{1}{\sqrt{P_{2}}}$.

The relationship between the control signal (diaphragm pressure) and the valve opening has its own dynamics. The system consists of a spring and, according to technical information provided by the manufacturer and the flow tests carried out at the Department of Mechanics in Turin Polytechnic (Italy) [15], it can be modeled as the second order system shown in Eq (7), where *K*_1_ is the gain, *ω*_*n*_ is the natural frequency, *ξ* is the absorption coefficient, Δ*A*(*s*) is the valve opening and Δ*U*(*s*) is the control signal (diaphragm pressure), both in Laplace domain.

$$q=417\cdot F_{p}\cdot P_{2}\cdot Y\cdot \sqrt{\frac{x}{G_{g}\cdot T\cdot Z}}\cdot \underset{C_{v}}{\underset{︸}{C_{v\;\;max}\cdot a}}$$ (1)

$$Y=1-\frac{x}{3\cdot F_{k}\cdot x_{T}}$$ (2)

$$\Delta P_{v}=P_{2}-P_{3}$$ (3)

$$x=\frac{\Delta P_{v}}{P_{2}}$$ (4)

$$F_{\gamma }=\frac{\gamma }{1.4}$$ (5)

$$\frac{q(t)}{a(t)}=C_{1}\cdot \sqrt{\Delta P_{v}\left(t\right)}+C_{2}\cdot {\left(\Delta P_{v}\left(t\right)\right)}^{3/2}$$ (6)

$$\frac{\Delta A(s)}{\Delta U(s)}=\frac{K_{1}\cdot \omega_{n}^{2}}{s^{2}+2\xi \omega_{n}s+\omega_{n}^{2}}$$ (7)

#### Pressure loss modeling

There are pressure losses throughout the pneumatic circuit which are caused by pipe length, valves and accessories (tees, silencers and diameter changes). In fluid dynamics, the Darcy-Weisbach Eq (8), see [16], relates the pressure loss caused by friction along a given length of pipe to the average velocity of the fluid flow, where *L*/*D* is the ratio of the length to the diameter of the pipe, *ρ* is the density of the fluid, *v* is the mean velocity of the flow, and *f* is the dimensionless Darcy friction factor. The velocity *v* can be expressed as in Eq (9), and the air volume flow *q* can therefore be related to the pressure loss.

Pressure losses in valves and accessories are usually calculated by using the Darcy-Weisbach equation and taking an equivalent pipe length (*L*/*D*)_*eq*_. Eq (10) is used to obtain the pressure loss between the compressor and the pneumatic silencers inside the HC, which is the sum of the pipe losses, valve losses and accessory losses. Since the other terms in the equation are constant, the pressure loss is equal to a constant *K*_2_ multiplied by *q*^2^, where it is assumed that a pressure change is proportional to the square flow [17].

$$P=f\cdot \frac{L}{D}\cdot \frac{\rho v^{2}}{2}$$ (8)

$$v=\frac{4}{\pi D^{2}}\cdot q$$ (9)

$$P_{1}-P_{3}=\sum \Delta P_{p}+\sum \Delta P_{v}+\sum \Delta P_{a}=K_{2}\cdot q^{2}$$ (10)

where Δ*P*_*p*_ belong to pipes, Δ*P*_*v*_ belong to valves and Δ*P*_*a*_ belong to other accessories.

#### Chamber pressurization modeling

If the air lost by the chamber is considered to be null, because it is not significant in comparison with the pressurization flow, the volumetric material balance is expressed by (11), where Δ*Q* is the mass flow (kg/s), *V* is the volume of the chamber (*m*^3^), and *ρ* is the air density (*kg*/*m*^3^). Due to the range of pressures during the treatments, the air inside the chamber can be considered as ideal air. The gas state equation is therefore shown in (12), where *R*_*S*_ is the specific gas constant $\left(\frac{m^{3}\cdot kPa}{K\cdot mol}\right)$, *T*_0_ is the temperature of the air (Kelvins), and Δ*P* is the increase in pressure inside the chamber (kPa). By combining Eqs (11) and (12), Eq (13) is obtained.

$$\Delta Q=V\frac{d\rho }{dt}$$ (11)

$$\frac{d\rho }{dt}=\frac{1}{R_{S}T_{0}}\cdot \frac{d\Delta P}{dt}$$ (12)

$$\Delta Q=\frac{V}{R_{S}T_{0}}\cdot \frac{d\Delta P}{dt}$$ (13)

#### Linearization of the chamber model

Next, the linearization of the main chamber is carried out and the structure of the transfer function (number of zeros, poles and integrators) is obtained. The highly non linear equations presented in the mathematical modeling above will be used only to know the structure of the linear system that better fits to the real behavior.

The term Δ*P*_*v*_(*t*) shown in Eq (6) is variant with time, because *P*_2_ and *P*_3_ are not constant. Eq (6) shows the relation between the opening of the valve and the volumetric fluid. If *P*_3_ is considered zero and *P*_2_ is considered constant, the equation would be a gain: $\frac{q(t)}{a(t)}=K_{3}$. Although Eq (6) is a non-linear equation, it has been linearized in order to obtain a transfer function. The equilibrium point is *q*_0_ = 0 (*q*(*t*) = *q*_0_ + Δ*q*(*t*)), *a*_0_ = 0 (*a*(*t*) = *a*_0_ + Δ*a*(*t*)) and Δ*P*_*v*_ = *P*_2_ = 10 bar (constants). The linearized expression of Eq (6) is shown in Eq (15).

The linearization leads to a lack of information because the *P*_2_ and *P*_3_ changing effects are now missing, but this approximation is necessary to obtain a linear model that will be validated in next sections. The Laplace transformation is used to obtain the transfer function shown in Eq (16). This equation shows a linear approximation of the behavior between the valve opening Δ*A*(*s*) and the fluid flow Δ*Q*(*s*). Since this expression is a gain, it is possible to merge it with Eq (7) in a single transfer function that represents the model of the control valve (17), which is a second order system where *K*_4_ = *K*_3_ ⋅ *K*_1_.

Eq (18) is obtained by linearizing Eq (10), while Eq (19) is obtained by applying the Laplace transformation to Eq (18). Similarly, applying the Laplace transformation to Eq (13) and considering *T*_0_ constant (isothermal process), the transfer function shown in Eq (20) is obtained.

Using Eqs (17), (19) and (20), the global transfer function in Eq (21) is obtained, which relates the pressure inside the chamber Δ*P*(*s*) with the valve control signal Δ*U*(*s*). Note that, the linear approximation of the system consists of one zero and three poles, one of which is an integrator.

$$P=P_{4}-P_{0}$$ (14)

$$\frac{\Delta q(t)}{\Delta a(t)}=C_{1}\cdot \sqrt{P_{2}}-C_{2}\cdot {\left(P_{2}\right)}^{3/2}=K_{3}$$ (15)

$$\frac{\Delta Q(s)}{\Delta A(s)}=K_{3}$$ (16)

$$\frac{\Delta Q(s)}{\Delta U(s)}=\frac{K_{4}\cdot \omega_{n}^{2}}{s^{2}+2\xi \omega_{n}s+\omega_{n}^{2}}$$ (17)

$$\Delta P_{1}-\Delta P_{3}=\underset{constant}{\underset{︸}{K_{2}\cdot 2\cdot q_{0}}}\cdot \Delta q=K_{5}\cdot \Delta q$$ (18)

$$\Delta P_{1}\left(s\right)-\Delta P_{3}\left(s\right)=K_{5}\cdot \Delta Q\left(s\right)$$ (19)

$$\frac{\Delta P(s)}{\Delta Q(s)}=\frac{\frac{R_{0}T_{0}}{V}}{s}=\frac{K_{6}}{s}$$ (20)

$$\frac{\Delta P(s)}{\Delta U(s)}=\frac{K_{5}\cdot K_{4}\cdot \omega_{n}^{2}\cdot s+K_{6}\cdot K_{4}\cdot \omega_{n}^{2}}{s\cdot (s^{2}+2\xi \omega_{n}s+\omega_{n}^{2})}$$ (21)

#### Experimental identification of the chamber model

The multiplace chamber considered in this work has three compartments and the volume of the HC in use is a combination between them depending on the doors open/closed, see Fig 3. Hence, the chamber volume can be either the volume of: the main chamber; the main chamber and pre-chamber; the main chamber, pre-chamber and ICU chamber; pre-chamber and ICU chambe; etc. In this section, the main chamber identification is shown, but the process has been performed to all combinations to obtain all possible models. A non-linear and a linear method are used for the identification.

**Fig 5:**
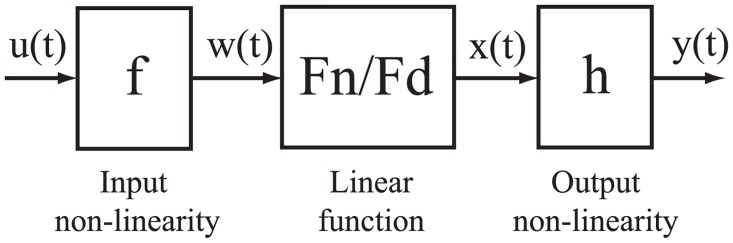
Structure of a Hammerstein-Wiener Model.

Non-linear identification. The most significant non-linearities of the system are given by (6), (10) and (13). A Hammerstein-Wiener Model [18] is considered for the non-linear identification. This model uses a linear dynamic transfer function and captures the non-linearities by using input and output non-linear static functions, see Fig 5, where: ‘f’ and ‘h’ are the input and output non-linear static functions, resepectively and ‘Fn/Fd’ is the linear dynamic transfer function, where ‘Fn’ and ‘Fd’ are polynomials of the linear output-error model. On the one hand, the linear transfer function is set to one zero and three poles (one integrator), see (21). On the other hand, both the input and output non-linear estimators are set with a piecewise linear function that is parameterized by 8 breakpoint locations, using a trial and error procedure. The number and location of those 8 breakpoints are decided due to the non linearity of the system, whereas the trial and error procedure is based on adjusting those parameters until the error is lower than the desired value of 0.05 bar.

Linear identification. The transfer function (21) is considered for the Linear Time-Invariant (LTI) Model.

### Controller for the chamber pressure

#### Description of the RST controller

**Fig 6:**
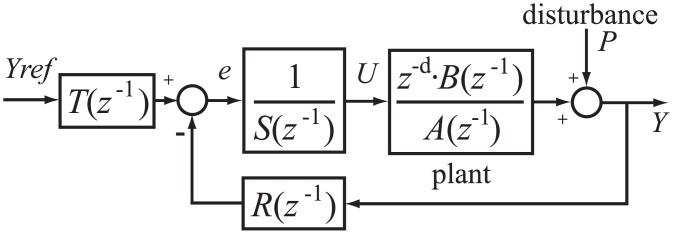
RST-controller structure.

In this work, a RST controller [19] has been designed and implemented to perform pressurization and depressurization profiles. This combination of feedforward and feedback digital controller is becoming a standard for computer control in industry [20]. Its general structure for linear single-input single-output systems is represented in Fig 6 where *R*, *S* and *T* polynomials have to be determined by zero-pole placement. The control command is calculated as can be seen below:

$$\begin{array}{c} U\left(z\right)=\frac{T(z^{-1})}{S(z^{-1})}\cdot Y_{ref}\left(z\right)-\frac{R(z^{-1})}{S(z^{-1})}\cdot Y\left(z\right) \end{array}$$ (22)

The RST control law is obtained following the next steps [21]:

1. Selection of the desired closed-loop transfer function as it is shown below:
  $$\frac{z^{-d}\cdot B\left(z^{-1}\right)\cdot T\left(z^{-1}\right)}{A\left(z^{-1}\right)\cdot S\left(z^{-1}\right)+z^{-d}\cdot B\left(z^{-1}\right)\cdot R\left(z^{-1}\right)}=\frac{B_{m}\left(z^{-1}\right)}{A_{m}\left(z^{-1}\right)}$$ (23)
  The structure of *A*_*m*_(*z*^−1^) is chosen as follows:
  $$\begin{array}{c} A_{m}\left(z^{-1}\right)=P_{dom}\left(z^{-1}\right)\cdot P_{aux}\left(z^{-1}\right) \end{array}$$ (24)
  where a group of dominant poles and auxiliary poles are selected.
2. Cancellation of stable poles and zeros of the process:
  The transfer function of the plant *H*(*z*^−1^) can be expanded to represent stable and unstable poles and zeros as it can be seen below:
  $$\begin{array}{ccc} H(z^{-1}) & = & \frac{z^{-d}\cdot B\left(z^{-1}\right)}{A(z^{-1})}\\ & = & \frac{z^{-d}\cdot B^{-}\left(z^{-1}\right)\cdot B^{+}\left(z^{-1}\right)}{A^{-}\left(z^{-1}\right)\cdot A^{+}\left(z^{-1}\right)} \end{array}$$ (25)
  where *A*^−^(*z*^−1^) and *B*^−^(*z*^−1^) contain the poles and zeros that cannot be cancelled [21]. For this reason, *B*^−^(*z*^−1^) cannot be a factor of *A*(*z*^−1^) ⋅ *S*(*z*^−1^) + *B*(*z*^−1^) ⋅ *R*(*z*^−1^). Thus *B*_*m*_(*z*^−1^) must be of the form *B*_*m*_(*z*^−1^) = *B*^−^(*z*^−1^) ⋅ *B*_*m*1_(*z*^−1^). The pole-zero cancellation yields:
  $$R(z^{-1})=A^{+}\left(z^{-1}\right)\cdot R_{1}\left(z^{-1}\right)$$ (26)
  $$S(z^{-1})=B^{+}\left(z^{-1}\right)\cdot S_{1}\left(z^{-1}\right)$$ (27)
  $$T(z^{-1})=A^{+}\left(z^{-1}\right)\cdot B_{m1}\left(z^{-1}\right)$$ (28)
3. Perturbation rejection in steady state: an appropriate number of integral terms are introduced in the control loop through polynomial *S*(*z*^−1^) as follows:
  $$\begin{array}{c} S_{1}\left(z^{-1}\right)={\left(1-z^{-1}\right)}^{n-l}\cdot S_{2}\left(z^{-1}\right) \end{array}$$ (29)
  where *n* is the perturbation order and *l* is the plant order.
4. Computation of polynomials *R*(*z*^−1^) and *S*(*z*^−1^):
  $$\begin{array}{cc} & A^{-}\left(z^{-1}\right)\cdot {\left(1-z^{-1}\right)}^{n-l}\cdot S_{2}\left(z^{-1}\right)+\\ & +B^{-}\left(z^{-1}\right)\cdot R_{1}\left(z^{-1}\right)=\frac{A_{m}\left(z^{-1}\right)}{A^{+}\left(z^{-1}\right)\cdot B^{+}\left(z^{-1}\right)} \end{array}$$ (30)
  where *R*(*z*^−1^) and *S*(*z*^−1^) are given by Eqs (26), (27) and (29).
5. Computation of *T*(*z*^−1^) using Eq (28). To obtain unit gain in the closed loop, condition *T*(1) = *R*(1) must be satisfied.

The transfer function of the whole system results in:

$$\begin{array}{cc} \frac{Y(z)}{Y\;ref(z)} & =\frac{z^{-d}\cdot B\left(z^{-1}\right)\cdot T\left(z^{-1}\right)}{A\left(z^{-1}\right)\cdot S\left(z^{-1}\right)+z^{-d}\cdot B\left(z^{-1}\right)\cdot R\left(z^{-1}\right)}\\ & =\frac{z^{-d}\cdot B^{-}\cdot B_{m1}}{A^{-}\cdot S_{1}+z^{-d}\cdot B^{-}\cdot R_{1}} \end{array}$$ (31)

For more details about the RST controller theory see [21].

#### Calculation of the RST controller

The RST theory described in previous subsection can be adapted and applied to HCs, where smoothness is the most important factor to be considered from the patient comfort point of view. Based on the knowledge of process dynamics and after a set of trials, a rising time of 6 seconds and a maximum overshoot of 0.05% have been chosen. Parameters *ω*_*n*_ and *ζ* of the continuous second order system are then obtained. Finally, the model is discretized using a sample time of 0.1 seconds.

To ensure the stability of the controller, the following robustness specifications are chosen for the gain and phase margins: Δ*M* ≥ 0.5 (−3*dB*) and Δ*τ* ≥ *T*_*S*_. In addition, a null or very low steady-state error must be guaranteed for ramp and parabolic inputs. Although in this case, three integrators would be required to get null steady-state error (parabolic input), a first calculation introducing only two integrators is performed to check the order of magnitude of the resulting error.

Regulation requirements and robustness specifications are summarized below:

- Dominant poles corresponding to the discretization of a second order continuous system with a rise time of 6 seconds and maximum overshooting of 0.05% (*ω*_*n*_ = 0.49 *rad*/*s* and *ζ* = 0.9):
  $$\begin{array}{c} P_{dom}\left(z^{-1}\right)=1-1.9125z^{-1}+0.9148z^{-2} \end{array}$$
- Gain margin: Δ*M* ≥ 0.5 (−3*dB*) and phase margin: Δ*τ* ≥ *T*_*S*_.
- Introduction of two integrators (see (29)): *S*_1_(*z*^−1^) = (1−*z*^−1^)^2^ ⋅ *S*_2_(*z*^−1^)
- Controller sample time *T* = 0.1 seconds.

After establishing the dynamic requirements, the controller can be calculated following the steps described above. Solving Bezout’s identity using Matlab function *bezout*.*m*, the following equations are considered:

$$\begin{array}{cc} A(z^{-1}) & \cdot B^{+}\left(z^{-1}\right)\cdot S_{1}\left(z^{-1}\right)+\\ & +z^{-d}\cdot B\left(z^{-1}\right)\cdot A^{+}\left(z^{-1}\right)\cdot R_{1}\left(z^{-1}\right)\\ & =P_{dom}\left(z^{-1}\right)\cdot P_{aux}\left(z^{-1}\right) \end{array}$$ (32)

T(1)=R(1) (33)

where *S*_1_(*z*^−1^) contains two integrators and the polynomials *A*(*z*^−1^) and *B*(*z*^−1^) are given by (39) and (38), respectively.

**Fig 7:**
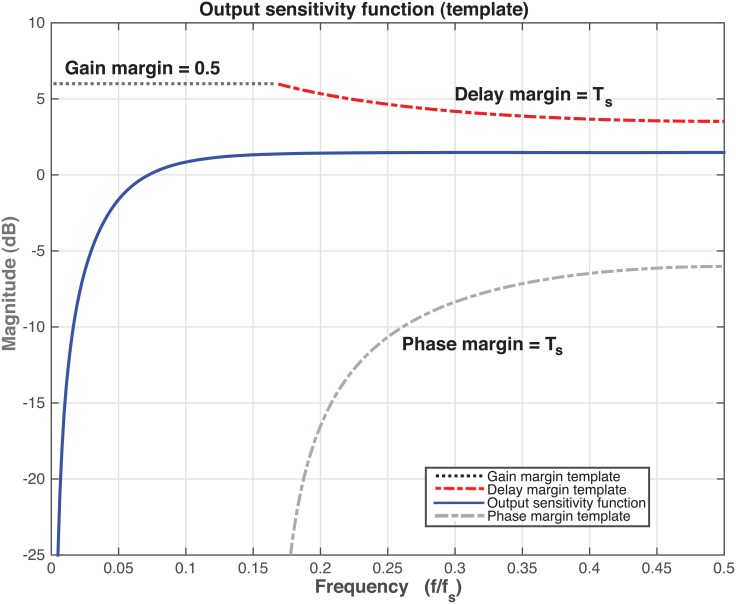
Output sensitivity function for the controller with two integrators and with auxiliary poles at *z* = 0.6.

Now, the auxiliary poles *P*_*aux*_(*z*^−1^) = (1 − 0.6*z*^−1^)^2^ are added to the controller in order to accomplish the robustness requirements. The output sensitivity function for this controller is shown in Fig 7, where it can be seen that the robustness specifications are met.

**Fig 8:**
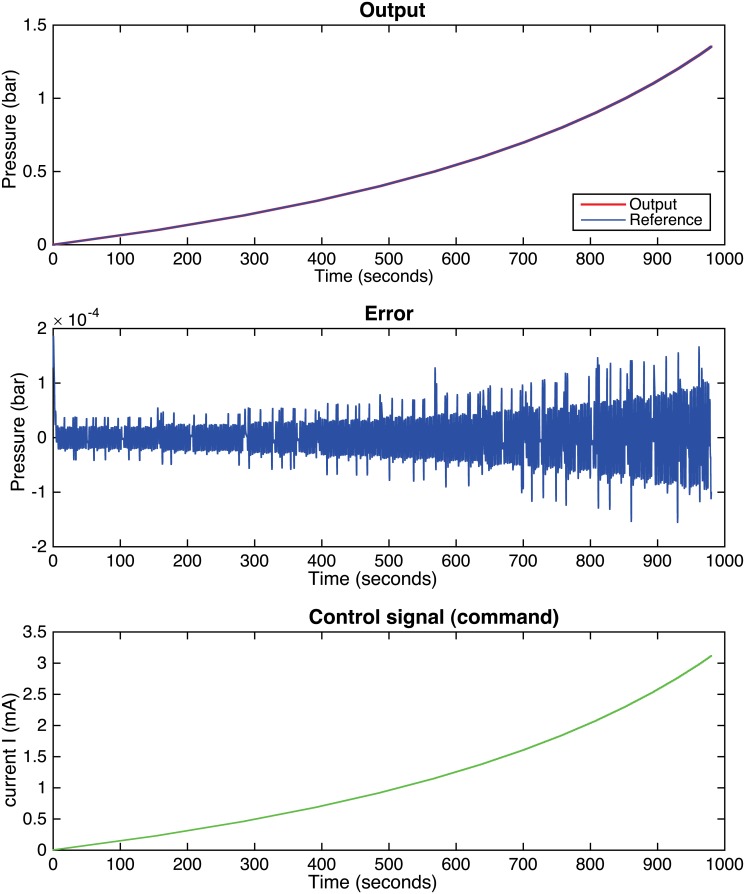
System response for a parabolic-type input and a two integrators controller with auxiliary poles in *z* = 0.6.

In this work, the HC uses a parabolic pressurizing profile as suggested in [22]. Fig 8 shows a simulation with this treatment profile. The steady-state error is not null because the controller has been designed for a ramp-type input (two integrators). Anyway, the error is really small: the absolute error is less than 2 ⋅ 10^−4^ bar at all samples and the average percentage error is around 0.01%.

Even tough the integration process has major benefits to control systems by eliminating steady state and even dynamic errors, there are stability issues that must be dealt with in the control system design process [23]. For this reason and due to the very low error obtained by the two integrators control law, this is the option implemented in this work.

**Fig 9:**
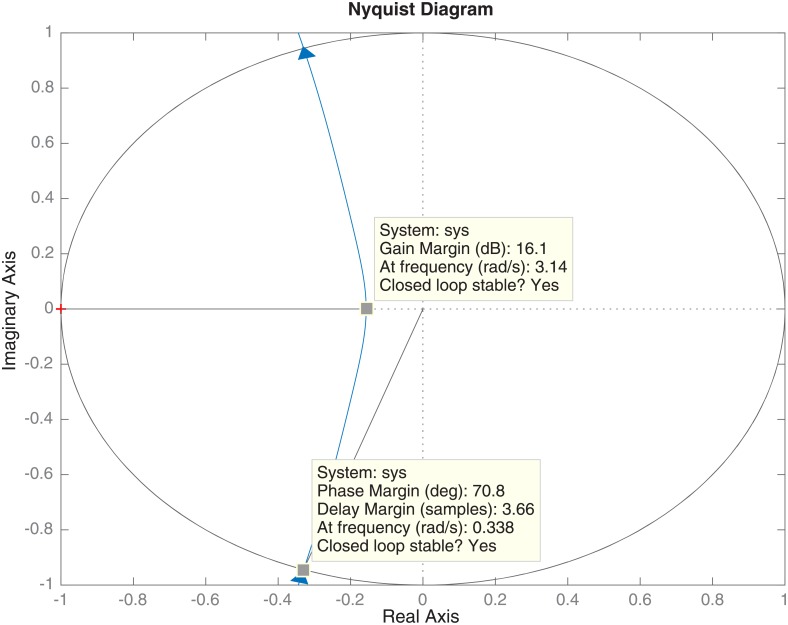
Nyquist diagram for the two integrators controller with auxiliary poles in *z* = 0.6.

In addition to the analysis of the output sensitivity function, which guarantees the robustness of the designed controller, the gain margin and the phase margin are analyzed by the Nyquist diagram in Fig 9. The Nyquist diagram yields a gain margin of 16.1 dB and a phase margin of 70.7 degrees. These values are within the typical robustness values. Moreover, there is a delay of 3.36 seconds, fulfilling the expression Δ*τ* ≥ *T*_*S*_ required in the controller design. Finally, using Eqs (26)–(28), the controller is calculated giving the following polynomials:

$$\begin{array}{c} R\left(z^{-1}\right)=31.71-74.32z^{-1}+55.09z^{-2}-12.44z^{-3} \end{array}$$ (34)

$$\begin{array}{c} S\left(z^{-1}\right)=1-2z^{-1}+1z^{-2} \end{array}$$ (35)

$$\begin{array}{c} T(z^{-1})=0.0367 \end{array}$$ (36)

### Conditions for the real experimentation

The input and output of the controlled system are the input current to the control valve (mA) and the pressure inside the chamber (bar), respectively. The sampling period for the experiments is set to one second. Moreover, prior to use, the system was adapted to meet the medical device regulations as described in the Discussion section.

#### Conditions for the identification tests

The *Matlab System Identification Toolbox* will be used for the identification process.

The input signal used for the identification (training data) consists of a white noise signal (i.e., a random signal with a constant distribution in the specified interval) since it allows to fit the model parameters accurately and within a wider frequency range than using steps as input, see [24]. Furthermore, for completeness, two different input signals are considered to validate the identified model (validation data): a white noise signal different to that used as training data and the classical step signal. The interval used for the white noise signals is [0 mA, 3 mA], whereas the step size used for the step signal is 7 mA.

For comparison purposes, the identification and validation results are expresed in terms of the Best Fit rate [25], which is defined as a percentage in Eq (37), where *y* is the measured output, $\hat{y}$ is the simulated model output, and $\bar{y}$ is the mean of *y*.

$$\begin{array}{c} \text{Best}\;\text{Fit}=(1-\frac{\|y-\hat{y}{\|}_{2}}{\|y-\bar{y}{\|}_{2}})\times 100 \end{array}$$ (37)

#### Conditions for the control tests

In order to show the ability of the designed chamber controller to accurately track complex pressure profiles, a real hyperbaric therapy will be carried out and the following quantitative measures of fit will be analyzed: maximum and root-mean-square (RMS) control errors. Moreover, the smoothness of the control signal is also an important factor, because the noise and air flows inside the chamber are minimized. For this purpose, the repeatability of the control experiment is analyzed by means of the standard deviation of the output pressure for ten experiments under the same conditions.

Furthermore, an illustrative clinical case will be considered to show the ability of the chamber controller to perform Pause and Alleviation procedures during the treatment.

## Results

### Identification results

For the identification test it has been obtained that the LTI model fits the training data by 96.81%, while the Hammerstein-Wiener model fits it by 99.52%. For the validation test using the white noise input signal, the LTI model fits by 93.93% and the Hammerstein-Wiener model fits by 95.79%. Furthermore, for the validation test using the step input signal, the LTI model fits by 98.35% and the Hammerstein-Wiener model fits by 98.48%.

**Fig 10:**
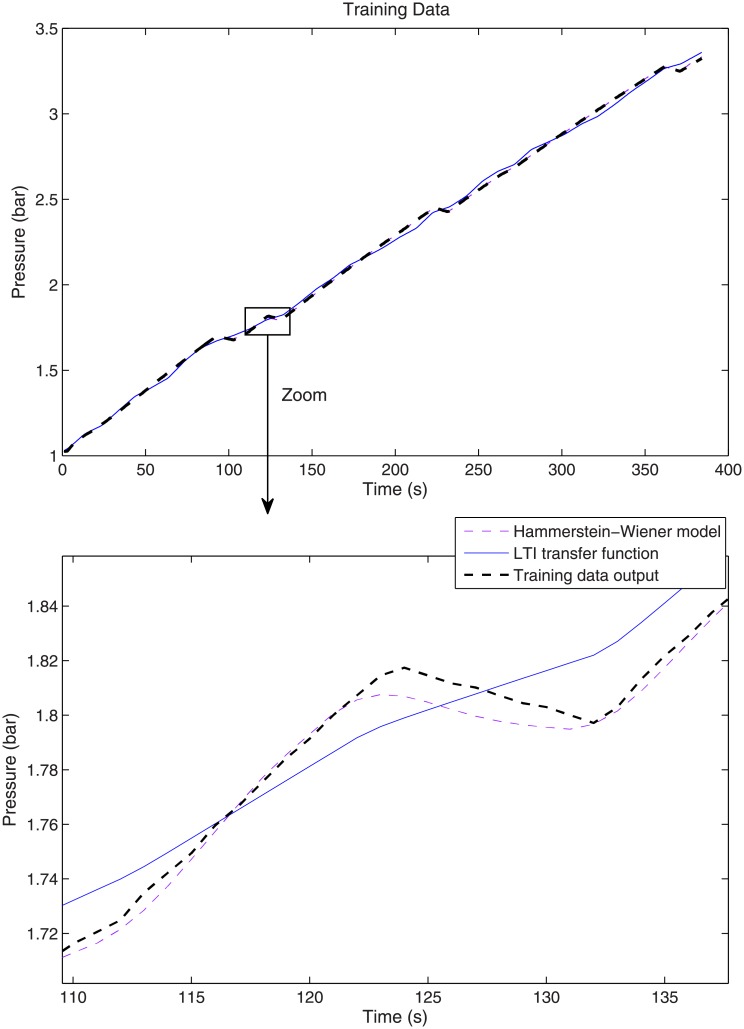
Training data results.

**Fig 11:**
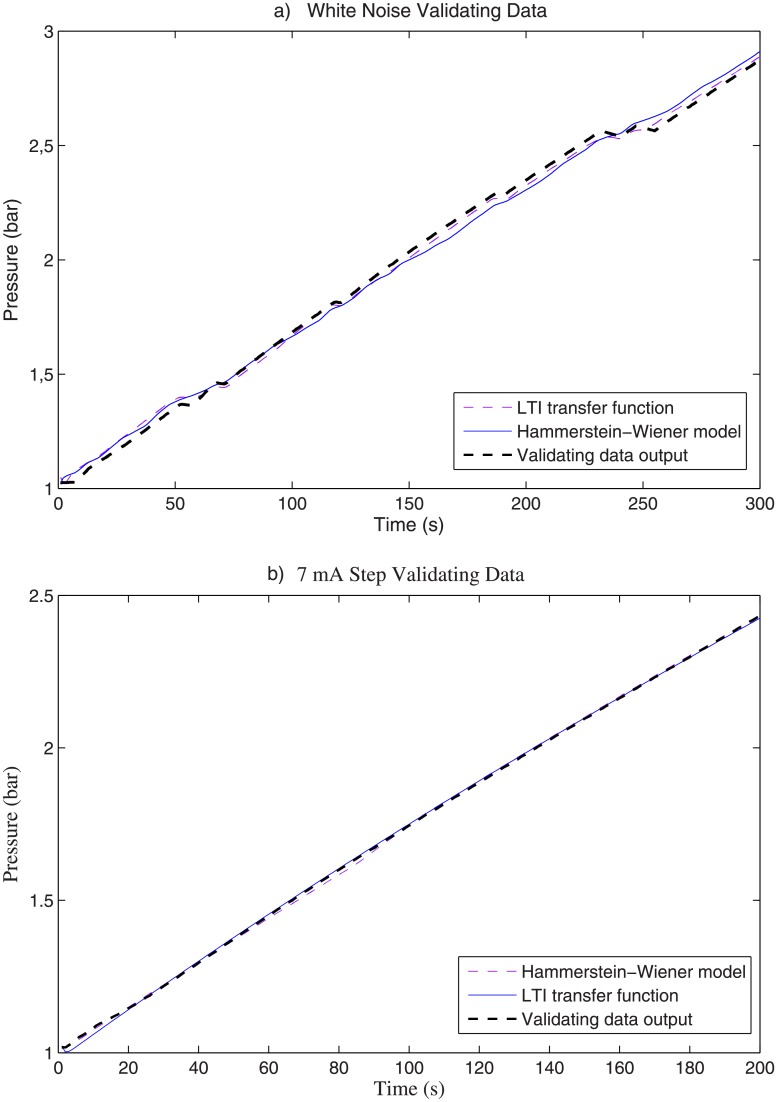
Validating data results. A: White noise. B: 7 mA Step.

Figs 10 and 11 show a comparison of the training and validation results, respectively, for the Hammerstein-Wiener and LTI models. The zoom image in Fig 10 (bottom) shows that the Hammerstein-Wiener Model is able to model the non-linearities better than the LTI model. Fig 11 shows the results using a different input signal (white noise signal and step) in order to validate the identified models.

It is worth mentioning that the process model includes an *integrator*, see Eqs (20) and (21), and hence the output pressure obtained for both the white noise input and the step input looks like a *ramp*, see Figs 10 and 11. That is, the positive value of the input signal, either the white noise or the step, is integrated and, thus, the output pressure is constantly increasing.

Although the Hammerstein-Wiener Model has obtained better results in the validation process, the identified LTI transfer function is accurate enough and is used to design the RST controller. The numerator and denominator polynomials for this transfer function are given by:

$$B(z)=-0.0001879+0.01025z^{-1}$$ (38)

$$A(z)=1-1.431z^{-1}+0.4542z^{-2}$$ (39)

### Experimental results for the chamber controller

**Fig 12:**
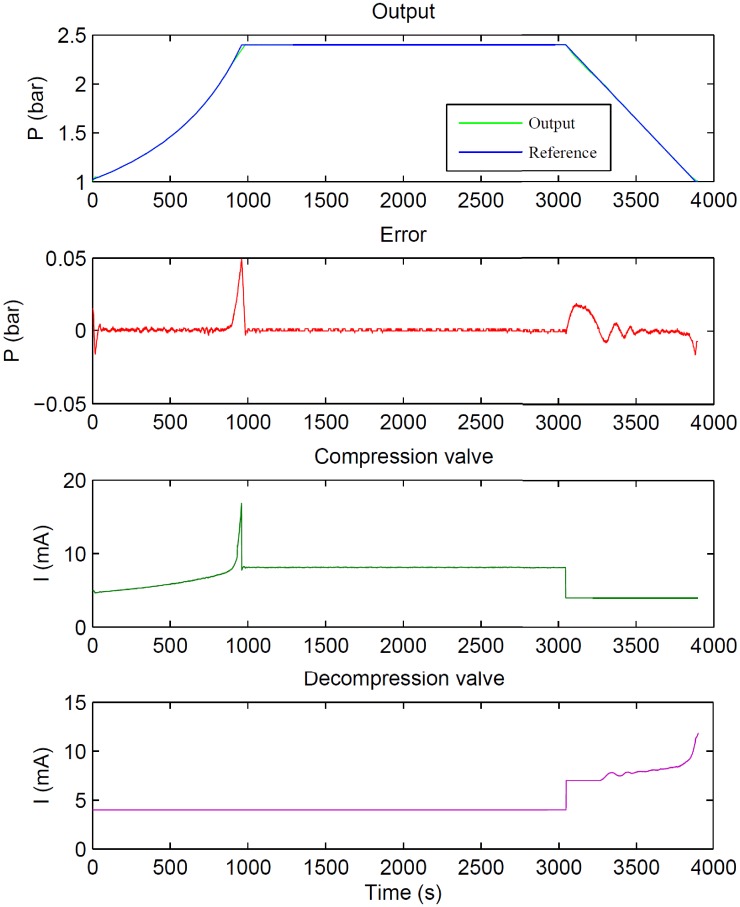
Real experiment for a treatment pressure profile using the designed controller.

Fig 12 shows a real hyperbaric therapy carried out with 25 patients. As can be seen, the pressure inside the chamber follows the profile accurately. In particular, the maximum and RMS control errors are around 0.05 bar (2.03%) and 0.006 bar (0.35%), respectively. Furthermore, the repeatability obtained with the automated chamber is good: the standard deviation of the output pressure for ten experiments under the same conditions was 0.01 bar (0.58%).

**Fig 13:**
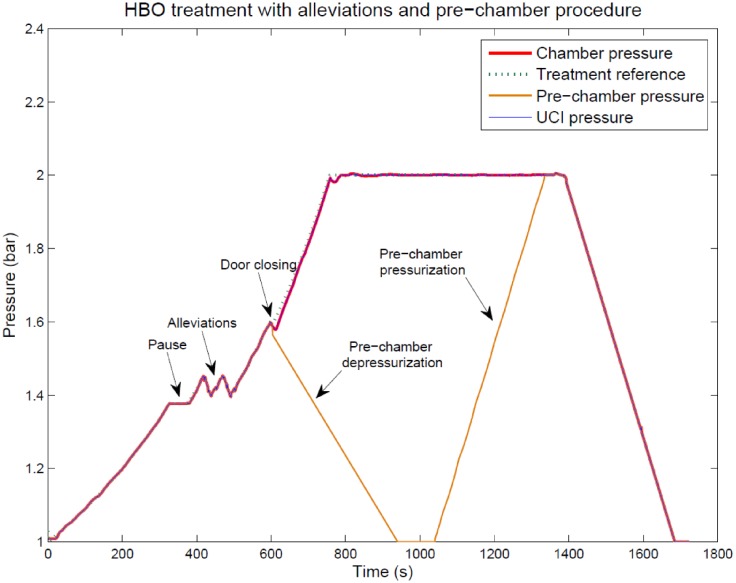
Real experiment for alleviate the chamber pressure and the pre-chamber procedure.

To illustrate how the system works, a clinical case is explained below. Fig 13 shows the chamber pressures during a real treatment in which the proposed control system is used. One of the patients had difficulties to compensate the pressure changes because of Eustachian tube obstructions (the patient was cold few days ago). Firstly, a Pause was performed in order to decide whether the patient could continue his treatment or not (the patient was suffering earache). Once the patient felt fine again, the pressure profile kept on pressurizing until the patient required to alleviate the pressure twice, so he could compensate the ear volume change. After both pressure reductions the treatment continued, but the patient continued complaining about earache so the clinical staff decided to take him out of the chamber. In that moment, the patient was placed inside the pre-chamber and its doors closed. When the control program detected the doors closing, a depressurization profile was loaded to the pre-chamber controller and as it can be seen in Fig 13 (orange line), the pre-chamber pressure was reduced to atmospheric pressure. While the pre-chamber procedure, the ICU and main chamber were separated and each regulator tracked the previously planned pressure profile. The patient was evacuated and a new pressurization profile loaded to take the pre-chamber to the ICU and main chamber pressure. When the pre-chamber reached 2 bar, its doors were opened and the whole chamber controller continued with the pressure profile. When the treatment was finished, the depressurization step took the pressure to the atmospheric level.

## Discussion

### Improvement obtained with the proposed approach

This work is focused on the automation of a HC. Although an automatic controlled machine presents obvious advantages, no clinical data collection has been rigorously conducted for comparison before and after the automation and, hence, the advantages listed below are kept as hypothetical only.

The control system presented in this work could help to reduce ear barotraumas as discussed below. Some technical reports [22] and chamber manufacturers [26] highlight the advantages of pressuring using a parabolic profile, namely *smooth ride*, in order to reduce the middle ear barotrauma. Although these data have not been published in peer review journals, if they are correct the best procedure to accurately generate this parabolic profile, or any other to be considered, is using a closed loop control system like the one presented in this work.

In general, the good repeatability achieved by the automated chamber, see the Results section, will allow the medical staff an accurate use of the chamber in the treatments without or with little experience.

Another advantage obtained with the developed automated chamber with respect to the same chamber manually controlled prior to automation could be the patient’s perception of comfort in terms of smoothness and low noise during the pressurization process.

Furthermore, a key advantage of the proposed automated control system with respect to the same chamber manually controlled prior to automation is the possibility to carry out automated complex procedures (see the illustrative clinical case in the Results section) without compromising the robustness and smoothness of the chamber pressure control and, hence, guaranteeing patients safety.

### Generalization of the procedure used for chamber automation

The mathematical *model* presented in this research applies to any HC regardless of its size and configuration (monoplace/multiplace and mono-chamber/multi-chamber), i.e., the modeling process remains the same and can be easily extrapolated. In addition, the *identification* procedure used in this work can be used as guideline to perform the identification of other HCs.

Regarding the *electromechanical scheme* used for the automation, which is shown in Fig 3, the configuration of the controller would remain as it is presented in this work regardless of the number of compartments, i.e., two programmable logic controllers (PLCs) working redundantly and a human machine interface (HMI). In addition, two pressure sensors and a temperature and humidity hair sensors are required per each compartment. To pressurize and depressurize each compartment, two sets of three valves in bypass configuration are also required. Each set of three valves is formed by an electrically actuated two inches valve, a manually actuated valve in parallel with the automatic one and another manually actuated valve in series with both.

### Medical device regulations

HCs in use are subjected to strict Medical Device (MD) regulations. In order to bring to market sanitary products in Europe, CE marking must be placed on the device. CE Marking on a product is a manufacturer’s declaration that the product complies with the essential requirements of the relevant European health, safety and environmental protection legislation. This conformity assessment is carried out by certification entities (e.g., SGS or TUV, amongst others) or sanitary authorities (e.g., European Medicines Agency). For this work, the regulation EN 14931:2007 about pressure vessels for human occupancy, Multi-place pressure chamber systems for hyperbaric therapy and performance, safety requirements and testing has been considered. However, further regulations have been taken into account, as discussed below.

The directives 90/385/EEC and 93/42/EEC are intended to harmonise the laws relating to MDs within the European Union. Manufacturers’ products meeting harmonised standards [27, 28] have a presumption of conformity to the directive and include the mentioned CE mark. The MD directive was reviewed and amended by the directive 2007/47/EC, which became mandatory on March 2010.

The list of MD premarket requirements can be found in [29]. In addition, the regulation RD-1591/2009 was followed during the design, implementation and validation of the automatic system presented in this work.

For those cases in which medical electrical equipment is used or a development of physiologic closed-loop controller is implemented, the regulation IEC 60601-1-10:2007 must be considered as well. This regulation includes the requirements for the development (analysis, design, verification and validation) of a physiologic closed-loop regulator in medical electrical equipment to control a physiologic variable. That is, the regulator modifies the controller output variable in order to adjust (i.e., change or maintain) the measured physiologic variable in order to track a reference value. However, the automatic control of a HC does not fit this case since its regulator controls an environmental variable, not a physiological variable of the patient.

## Conclusion

The hyperbaric chamber automated in this work is being used in a real hospital since 2015. From then, more than three thousand treatments have been successfully carried out. The control system has been also tested with special patients (e.g., patients with esophagus balloon or autistic) before publishing this work. The system automation has provided the following benefits:

- A good pressure profile tracking (average error equal to 0.35%) and repeatability (standard deviation equal to 0.58%) are obtained.
- Stability in pressurization during bottom treatment independently of disturbances, air escapes or use of medical locks are now compensated by the controller.
- Implementation of complex procedures during the chamber pressurization like Alleviation and Pause are now automatically performed by the system only pressing a button.
- Automatic procedures for pre-chamber depressurization and pressurization are now very simple.
- Calculation of treatment end is now automatically and accurately performed by the system. A treatment delay is computed by the system to accomplish initial specifications indicated by the doctor depending on Alleviations and Pauses introduced during the pressurization procedure.

Moreover, a clear procedure for monoplace and multiplace chamber modeling, identification, controller calculation and system implementation has been provided. Furthermore, the medical device regulations considered to automate the chamber have been also detailed. All this information can be useful to automate other types of hyperbaric chambers in order to get the listed advantages either for medical treatments or research activities.
